# The Reciprocal Influence of Acute Diseases and Menstruation

**Published:** 1852-10

**Authors:** 


*On the Reciprocal Influence of Acute Diseases and Menstruation.* By M. Herard.—M. Herard terminates a recent memoir with the following conclusions:—1. All acute diseases exert a pretty similar effect on menstruation.—2. This influence varies accordingly as the diseases become developed during a menstrual epoch, or during an interval.—3. In the first of these cases the menses are usually suppressed completely or incompletely, when they may reappear after some hours or days, though usually in diminished quantity. The patients regard the suppression as being the cause of the febrile disease, although the contrary is the fact: and even in the case of acute febrile disease becoming manifested after suppression, we must regard it as a consequence of the chill that has produced this.—4. When an acute febrile disease is developed in the interval, if the next epoch is near at hand, so that the fever continues to it, the menstruation is favored by the increased hæmorrhagic congestion of the uterus and ovaries.—5. The menses are usually absent or notably diminished in quantity, at the periods which occur during the decline of a disease, or in convalescence. This secondary amenorrhœa, though sometimes persistent, usually only continues for from one to three months.—6. The menstrual eruption in nowise predisposes to disease.—7. Menstruation exerts no appreciable influence on the issue of acute febrile affections. The progress and termination of these are the same, whether the discharge appears or not, whether it is increased or diminished in quantity, is earlier or later in appearance, or whether this takes place at the beginning or end of the affection.—8. In treating acute febrile affections, it is the condition of the disease that must engage our attention; for it is rare that any special therapeutical indication is derivable from the state of the menses; and we must act absolutely in the same way if the menses are on the point of appearing, or are expected, as if they were not so.—9. Bloodletting does not, in general, prevent their appearance or continuance—10. The sudden suppression of the menses by the development of an acute febrile disease, or amenorrhœa consecutive to such disease, does not, in general, call for any special treatment.—*L'Union Medicale*, 1851, No. 149.

